# Validity of Wrist-Worn Activity Trackers for Estimating VO_2max_ and Energy Expenditure

**DOI:** 10.3390/ijerph16173037

**Published:** 2019-08-22

**Authors:** Stefanie Passler, Julian Bohrer, Lukas Blöchinger, Veit Senner

**Affiliations:** Professorship of Sport Equipment and Materials, Department of Mechanical Engineering, Technical University of Munich, Boltzmannstraße 15, D-85747 Garching, Germany

**Keywords:** consumer wearable devices, validation, accuracy, sports watches, fitness trackers, monitoring, physical activity

## Abstract

Activity trackers are a simple and mostly low-priced method to capture physiological parameters. Despite the high number of wrist-worn devices, there is a lack of scientific validation. The purpose of this study was to assess whether the activity trackers represent a valid alternative to gold-standard methods in terms of estimating energy expenditure (EE) and maximum oxygen uptake (VO_2max_). Twenty-four healthy subjects participated in this study. In total, five commercially available wrist-worn devices were tested with regard to their validity of EE and/or VO_2max_. Estimated values were compared with indirect calorimetry. Validity of the activity trackers was determined by paired sample t-tests, mean absolute percentage errors (MAPE), Intraclass Correlation Coefficient, and Bland-Altman plots. Within the tested devices, differences in scattering in VO_2max_ and EE could be observed. This results in a MAPE > 10% for all evaluations, except for the VO_2max_-estimation of the Garmin Forerunner 920XT (7.3%). The latter significantly underestimates the VO_2max_ (t(23) = –2.37, *p* = 0.027), whereas the Garmin Vivosmart HR significantly overestimates the EE (t(23) = 2.44, *p* = 0.023). The tested devices did not show valid results concerning the estimation of VO_2max_ and EE. Hence, the current wrist-worn activity trackers are most likely not accurate enough to be used for neither purposes in sports, nor in health care applications.

## 1. Introduction

The results of a worldwide survey of fitness trends in 2019 have shown that wearable technology will continue to be number one [1]. This trend has been observed since 2016. However, many consumers are not aware of the fact that wearable devices sometimes make inadequate and inaccurate predictions regarding the measurement accuracy [2]. Nowadays, a wide range of consumer wearable devices are available. The term wearable devices, which is often abbreviated as wearables, usually refers to small computer-controlled systems that are worn in, on, and close to the body. They are often equipped with a variety of sensors (e.g. accelerometers, gyroscopes, magnetometers, pulse oximeters). These sensors enable the devices to collect information about their immediate environment and therefore to monitor physiological signals such as number of steps, heart rate, quality of sleep, sleep rhythm, energy expenditure (EE), and maximum oxygen uptake (VO_2max_) [2,3,4,5,6,7,8,9,10,11,12,13,14]. In the rapidly growing field of wearables, tattoos and subcutaneous implants have also become a subject of research. However, the best-known and most common wearable devices are wrist-worn activity trackers. They represent one of the simplest and most cost-effective ways of monitoring various physiological parameters. Despite the high number of wrist-worn activity trackers [14], there is a lack of scientific validation studies. Energy expenditure seems to be the most critically examined physiological parameter. In Evenson et al. [2], a systematic summary of the validity and reliability of activity trackers is provided. This review includes validation studies on activity trackers from the Fitbit and Jawbone brands. For EE, almost all trackers demonstrate good to excellent reliability (Intraclass Correlation Coefficient, ICC = 0.74–0.97). However, they do not provide valid results. Estimation of EE is usually significantly underestimated. Further studies confirm that a lot of wrist-worn activity trackers show insufficient validity and reliability concerning EE [3,4,5,6,7,9,10,11,13,15,16]. Boudreaux et al. [16] investigated the validity of EE during cycling and resistance exercise. Thereby, none of the tested devices showed valid results. Besides the general validity, Wahl et al. [15] examined the influence of running pace on the EE. The study shows a significant influence of running pace on the estimated EE. Energy expenditure tends to be overestimated at lower pace and underestimated at higher pace. Equally, Roos et al. [5] examined the validity of estimated EE during running. They concluded that metabolism significantly influences the estimation of EE. In the aerobic range, EE was both over- and underestimated, whereas in the anaerobic range, the tested sports watches significantly underestimated EE by 21.6% to 49.3%, respectively. Woodman et al. [3] also reported significant mean absolute percentage errors of up to 64% in EE. Concerning the VO_2max_ estimation of wrist-worn activity trackers, a small number of scientific validation studies have been published so far. To our knowledge, Kraft & Roberts [8] and Snyder et al. [17] are the only scientific validation studies concerning the prediction of the maximum oxygen uptake by means of activity trackers. Kraft & Roberts [8] tested the accuracy of VO_2max_ prediction of the Garmin Forerunner 920XT. Thereby, the sports watch does not show significant differences in comparison with spirometry. Snyder et al. [17] investigated the validity of VO_2max_ prediction of three sports watches—Polar V800 and Garmin Forerunner 230 and 235. They showed significant differences in comparison with the gold standard and observed a significant influence of gender. Based on the differing results of the above-mentioned validation studies, estimations of EE and VO_2max_ should be regarded with skepticism and caution. In general, these publications and their results underline the necessity for comprehensive scientific validation of wearable devices. 

Performance-specific misjudgments of activity trackers can lead to an increased risk of injury due to overload. Consumers must be protected, especially if activity trackers are to be used increasingly in the health sector and if they are granted increasing access to our society. However, this access can only be defended or considered responsible if the current lack of transparency of the activity trackers industry is remedied through high-quality research, which can also help define general standards for these devices. Henceforth, the interdisciplinarity of different fields, especially sports science, medical technology, and ergonomics, but also standardization, is in demand.

The aim of the study was to clarify the validity of wrist-worn activity trackers. Both, the prediction of the VO_2max_ and EE are physiological parameters used for training control in sports and for support of obesity treatment. Therefore, they are linked to both physical activity and healthy lifestyle. The purpose was to assess whether the activity trackers represent a valid alternative to the respective gold standard method in terms of predicting EE and VO_2max_ and whether they can be used without hesitation in the health sector or for training control in sports.

## 2. Materials and Methods 

### 2.1. Participants

Twenty-four healthy men (*N* = 13) and women (*N* = 11) agreed to participate in the study. The study was conducted in accordance with the Declaration of Helsinki. Participants who were eligible received detailed information on the purpose and methods of the study, as well as on data treatment and confidentiality according to the General Data Protection Regulation (2016/679) of the European Parliament and the Council of 27 April 2016 [18] and its Corrigendum of 23 May 2018 [19]. Based on this information, they provided written consent. In order to standardize the examination conditions, the following criteria were defined: The consumption of the last high-carbohydrate meal must have taken place at least 2 h ago; no caffeine-containing foods may be consumed 24 h before measurement; no intensive sports activities and no consumption of alcohol or other stimulants 24 h before measurement. In order to standardize the test procedure, participants had to pass a performance diagnostic test. These diagnostics were proceeded under medical supervision of the Department of Prevention, Rehabilitation, and Sports Medicine of the Technical University of Munich.

### 2.2. Activity Trackers Used in this Investigation

The following two sports watches, Polar V800 and Garmin Forerunner 920XT, and three fitness trackers, Garmin vivosmart® HR, TomTom Touch, and Withings Pulse O_x_, were investigated.

#### 2.2.1. Polar V800

Since early 2014, the Polar V800 (Polar Electro Oy, Kempele, Finland) has been a popular sport watch amongst professional and recreational athletes in various sports. The watch includes several sensors (accelerometer, gyroscope, barometer, GPS and Bluetooth), provides functions (e.g. track route, number of steps) and various sport profiles. For heart rate monitoring, the Polar V800 (PV800) uses a chest strap from the Polar H-Series in combination with firstbeat technology from Firstbeat Technologies Oy (Jyväskylä, Finland). If used with a compatible chest strap and with preliminary information about age, height, body weight, etc. the PV800 provides information on training status, training load, and recommended recovery time. Thereby, an orthostatic Schellong test protocol estimates the user’s VO_2max_, which is equivalent to training status. The sports watch can be connected to the manufacturers’ website or an App called Polar Flow (Polar Electro Oy, Kempele, Finland), where detailed information on e.g. training load and recovery can be analyzed.

#### 2.2.2. Garmin Forerunner 920XT

The Garmin Forerunner 920XT (Garmin International Inc., Olathe, Kansas, USA) is another popular sport watch amongst athletes of all kinds, but especially triathletes. Like the Polar V800, it includes a quite similar set of sensors, functions and sport profiles. For heart rate monitoring, the Garmin Forerunner 920XT (GF920XT) uses an external chest strap and first beat technology. If used with a compatible chest strap and with preliminary information about age, height, body weight, etc. the sports watch provides information on training status, training load, and recommended recovery time. In comparison with the PV800, the GF920XT does not rely on an orthostatic test for estimating VO_2max_. Instead, the GF920XT uses user information, e.g. heartrate, gathered during an outdoor run at comfortable, submaximal running pace. For the VO_2max_ estimation, this run has to last at least 10 minutes. The algorithm for VO_2max_ estimation is not known. The sports watch can be connected to the website or App Garmin Connect (Garmin International Inc., Olathe, Kansas, USA), where detailed information on e.g. training load and recovery can be analyzed.

#### 2.2.3. Garmin Vivosmart® HR

The fitness tracker Garmin vivosmart® HR (GVHR) (Garmin International Inc., Olathe, Kansas, USA) is worn on the wrist. The recorded parameters include number of steps, distance, heightened floors, energy expenditure, heart rate, and sleep monitoring. To calculate energy expenditure, the tracker uses anthropometric data as well as the user's heart rate. To record activity and thus energy expenditure, the activity log must be activated on the tracker. After synchronizing of the tracker and the Garmin Connect website or the App (Garmin International Inc., Olathe, Kansas, USA), activity can be viewed and analyzed.

#### 2.2.4. TomTom Touch

The fitness tracker TomTom Touch (TTT) (TomTom N.V., Amsterdam, The Netherlands) should be worn tightly above the wrist bone. It monitors number of steps, energy expenditure, active time, distance, heart rate, and body composition. To calculate energy expenditure, the tracker uses information about gender, weight, training intensity, training duration, and heart rate. In addition, so-called Metabolic-Equivalent-Tables are used for more detailed information [20]. These tables contain information on the metabolic equivalents of different types of physical activity. After synchronizing with TomTom My Sports (TomTom N.V., Amsterdam, The Netherlands), the collected information of the activity can be viewed and analyzed.

#### 2.2.5. Withings Pulse O_x_

The fitness tracker Withings Pulse O_X_ (Nokia Oyj [formerly Withings], Espoo, Finland) can be worn on the wrist or attached to the waistband of the trousers. It records data on number of steps, altitude, distance, energy expenditure, and sleep quality. The Withings Pulse O_x_ (WPO_x_) calculates energy expenditure based on anthropometric data and number of steps. In addition, a subjective assessment of the participants’ physical strain may lead to data that are more accurate. Thereby, participants should indicate their physical stress (In the context of this publication “strain“ is related to a load or loading profile the athlete is exposed to. “Stress” is the subjective and/or physiological response to this load) on a scale from one to five. 1 means no sweating; 2 corresponds to a slight burning of the muscles; 3 corresponds to an increased heart rate; 4 is defined as a very intensive physical strain; and 5 corresponds to full exhaustion. Data can be viewed via the Nokia Health Mate™ App (Nokia Oyj, Espoo, Finland) on the smartphone or via the Nokia Health Mate website.

### 2.3. The Gold Standard in this Investigation

As the gold standard to measure VO_2max_ and energy expenditure, the spirometry device Metalyzer 3B-R3 (Cortex Biophysics GmbH, Leipzig, Germany) and MetaSoft Studio Software (Cortex Biophysics GmbH, Leipzig, Germany) were used. Cortex Metalyzer 3B (CM3B) is a stationary respiratory gas analysis system using Breath-by-Breath technology. This technology enables a precise and accurate determination of the individual maximum oxygen uptake rate and the energy expenditure [21,22,23,24] and has thus been used in various scientific studies in sports medicine [3,6,7,8]. The estimation of energy expenditure is based on the ratio of inhaled oxygen to exhaled carbon dioxide. During the diagnostics, the participant has to wear a breathing mask that is connected via a tube to an analyzer. Respiratory gases of each breath are analyzed. 

### 2.4. Procedure

Participants fulfilled a questionnaire before running the first trial and for inclusion/exclusion of the study. Anthropometric questions, such as gender, age, height, and weight, were followed by 14 short questions on health status and physical activity. There were eight questions on cardiac and other physical problems to investigate the general fitness of the participants (Physical Activity Readiness (PAR)–Questionnaire, [25]). If one or more questions of the PAR-Questionnaire were affirmed, participants were excluded from the study. Moreover, there were two questions on endurance performance (Perceived Functional Ability (PFA)–Questionnaire, [26]), and four questions on the subjects' activity level over the last 3 to 6 months. The performance diagnostic was only carried out if health problems and pre-existing conditions could be excluded. The study was divided into a lab session (Prevention & Performance Lab of the Technical University of Munich) and a field test (400-Meter-Athletics track). Tests for both parameters had to be carried out separately because of their different test procedures.

During the first session, participants had to undergo performance diagnostics to determine their individual VO_2max_ by spirometry. The height and the body weight of the participants were determined using the electric floor scale Seca (SECA Germany, Hamburg, Germany) with an integrated stadiometer. After collecting preliminary anthropometric data for VO_2max_ estimation of the PV800, an orthostatic pretest (10 minutes of laying in supine position) was performed with the H-7 chest strap to determine resting heart rate, average heart rate and maximum heart rate. Afterwards the protocol for the main orthostatic VO_2max_ estimation of the PV800 was performed, including three minutes of lying in supine position and standing upright for another 3 minutes subsequently. In medical science, this test is often referred to as the Schellong test (usually both pulse and blood pressure are monitored during the procedure). In functional diagnostics, this method is commonly used to determine orthostatic dysregulations such as orthostatic hypotension, postural orthostatic tachycardia syndrome, and neurocardiogenic syncope [27]. The Schellong test was followed by the spirometry with CM3B on the treadmill Hp-Cosmos Pulsar (Hp-Cosmos sports & medical GmbH, Nußdorf, Germany). This performance diagnostic was used to determine the maximum oxygen uptake rate of the participants and to standardize the exercise intensity when measuring energy expenditure. During the diagnostic, participants were secured with a belt and a safety rope attached to the treadmill. A standardized load pattern, created in MetaSoft Studio Software (Cortex Biophysics GmbH, Leipzig, Germany), was used. According to Midgley et al. [28], a load duration of 5 to 26 minutes is sufficient for valid determination of VO_2max_. Because of this, a flat load pattern was chosen. As a result, measured parameters increase more slowly. In this way, the ventilatory thresholds and heart rate zones can be determined more precisely. The load pattern started with a 3-minute measurement at rest. Thereby, the participants have to stand on the treadmill without moving or talking, while breathing calmly. After this measurement at rest, a 3-minute warm-up took place, whereby the participants had to run at a speed of 6 km/h. Then the load pattern started at 7 km/h and increased continuously by half a km/h per minute. The running pace was increased until a leveling off of the participant’s oxygen consumption could be observed. 

During the second session, VO_2max_ estimation by the GF920XT (Garmin International Inc., Olathe, Kansas, USA) was proceeded. In addition, tests concerning the energy expenditure were carried out. 

#### 2.4.1. VO_2max_

For estimating VO_2max_ by the GF920XT, participants had to complete a field-endurance-run at self-chosen comfortable pace and for a duration of at least 10 minutes. Based on the preliminary information given and the information obtained during the run, the VO_2max_ was then estimated.

#### 2.4.2. Energy Expenditure 

During the second session, the energy expenditure was measured under submaximal load on the treadmill Hp-Cosmos Pulsar. The fitness trackers GVHR, TTT, and WPO_X_ were compared with the CM3B. The test was proceeded at running pace, reached at 55% and 70% of the individual VO_2max_. The fitness trackers were placed at the wrist as recommended by the manufacturer. The load pattern used for estimating EE of the fitness trackers consists of two steps. The first step lasted 5 minutes and served as a warm-up. Participants had to run at their individual speed, reached at 55% of VO_2max_. At the end of the warm-up, the treadmill was stopped for a few seconds to activate the activity protocol on GVHR and on TTT. In addition, the WPO_x_ was set up during this time. The second step of the load pattern then started. It lasted 10 minutes and was used to record energy expenditure. Participants had to run at their individual speed, reached at 70% of VO_2max_. Because of the fact, that indirect calorimetry can only provide valid comparative values at a submaximal load below 75% of VO_2max_ [29], this load pattern was chosen.

### 2.5. Data Analyses

Statistical analyses were conducted using IBM SPSS Statistics software version 24 (IBM, Armonk, New York). Descriptive statistics were used to characterize the sample population. The validity of the activity trackers was determined by several statistical tests. Data from sports watches and fitness trackers were compared with the results of spirometry using paired sample t-tests. An alpha of 0.05 was used to determine statistical significance. In addition, the mean absolute percentage errors (MAPE) were calculated as an indicator of measurement error. MAPE, representing the error as a percentage of the overall mean relative to the spirometry, does not have a standardized threshold for determining the validity of measurements. In the present study, a MAPE of ≤ 10% [6] was used as the criterion value for validity. According to Ranganathan et al. [30] and Liu et al. [31], the Intraclass Correlation Coefficient (ICC) defined the agreement between the gold standard and the tested devices. In general, this coefficient provides an estimate of overall concordance between two methods. The ICC indicates the between-method variability expressed as a proportion of the total variance of the results [32]. Excellent, good, moderate, and low agreement thresholds were defined as ICC values of ≥ 0.90; 0.75–0.90; 0.60–0.75; and ≤ 0.60, suggested by Fokkema et al. [12]. ICC is inter alia a commonly used method to validate wearable devices as previously used by Fokkema et al. [12], Wahl et al. [15], and Boudreaux et al. [16].

To investigate the level of agreement, Bland-Altman plots were prepared according to Bland & Altman [33]. For this, limits of agreement were set to 95%.

## 3. Results

Characteristics of the sample population are shown in Table 1.

### 3.1. VO_2max_

The results of the descriptive examination of the differences between the measured VO_2max_ (CM3B) and the estimated VO_2max_ of the investigated sports watches GF920XT and PV800 are provided in Table 2. 

On average, participants achieved a value of 50.3 ± 8.1 ml·kg^−1^·min^−1^ in spirometry. The average estimated VO_2max_ of GF920XT and PV800 is 48.1 ± 6.5 ml·kg^−1^·min^−1^ and 53.2 ± 10.5 ml·kg^−1^·min^−1^, respectively. The results of t-tests comparing VO_2max_ from sports watches with a spirometry device show significant underestimations by the GF920XT (t(23) = –2.37, *p* = 0.027), whereas the PV800 indicates no significant tendency to overestimate the VO_2max_ (t(23) = 1.89, *p* = 0.071). Even though both devices are quite similar in terms of mean absolute errors (MAE), a MAPE of 13.2% (PV800) and 7.3% (GF920XT) was determined, respectively. Moreover, GF920XT and PV800 show moderate to good agreement (ICC) in comparison with CM3B (GF920XT: 0.82; PV800: 0.67) and high internal differences in variance (GF920XT: 42.1; PV800: 109.6).

**Figure 1:**
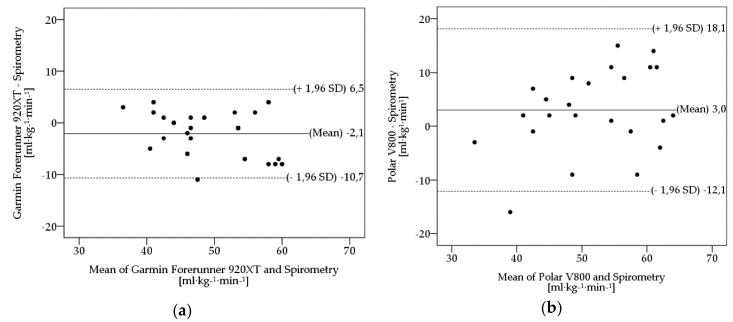
Bland-Altman plots of Spirometry VO_2max_ for (**a**) Garmin Forerunner 920 XT, (**b**) Polar V 800. Limits of agreement (LoA) were calculated as mean ± 1.96 x SD. Mean biases are depicted as solid line; LoA are depicted as dashed lines.

Figure 1 shows Bland-Altman plots of the sports watches PV800 and GF920XT in comparison with spirometry with CM3B. 

These plots serve as a visual illustration of scattering and over- or underestimated measurement ranges of the investigated sports watches. The plots indicate the differences of the VO_2max_ values on the y-axis relative to the mean of the two methods (spirometry and alternative method) on the x-axis. Mean differences (bias) between estimated VO_2max_ and VO_2max_ of spirometry, upper and lower limits of agreement (ULoA, LLoA) are labeled in the plots. Limits of agreement (LoA) were calculated as means ± 1.96 x SD. Both sports watches show considerable deviations in scattering, when compared with spirometry. The plots illustrate the PV800’s tendency to overestimate (bias: 3.0 ml·kg^−1^·min^−1^) and the GF920XT’s tendency to underestimate (bias: –2.1 ml·kg^−1^·min^−1^) the VO_2max_, respectively. Furthermore, the differences in variance are visualized. The PV800 (ULoA-LLoA: 30.2 ml·kg^−1^·min^−1^) shows higher scattering amongst its measures when compared with the GF920XT (ULoA-LLoA: 17.2 ml·kg^−1^·min^−1^).

### 3.2. Energy Expenditure

The results of the descriptive examination of the differences between the measured EE (CM3B) and the estimated EE of the investigated fitness trackers TTT, GVHR, WPO_x_ with and without the adjustment of the subjective assessment of the participants’ physical strain are provided in Table 3.

On average, participants achieved an EE of 125.5 ± 35.3 kcal in spirometry. The average estimated EE of TTT is 130.0 ± 23.2 kcal, of GVHR is 139.8 ± 28.8 kcal, of WPO_x_ without adjustment is 121.8 ± 24.4 kcal, and of WPO_x_ with adjustment is 121.5 ± 22.0 kcal, respectively. Based on the results of the t-test, the GVHR significantly overestimates the EE (t(23) = 2.44, *p* = 0.023). The fitness trackers TTT (t(23) = 0.93, *p* = 0.363), WPO_x_ without adjustment (t(23) = –0.54, *p* = 0.590), and WPO_x_ with adjustment (t(23) = 0.90, *p* = 0.377) indicated no significant tendency to underestimate or overestimate the EE.

This results in an MAPE of 18.2% (TTT), 23.9% (GVHR), 20.1% (WPO_x_ without adjustment), and 14.2% (WPO_x_ with adjustment), although the MAE of the fitness trackers, except for the GVHR, were very similar. Moreover, the fitness trackers show low to moderate agreement (ICC) in comparison with CM3B (TTT: 0.68; GVHR: 0.60; WPO_x_ without adjustment: 0.40; WPO_x_ with adjustment: 0.72) and exhibit high internal differences in variance (TTT: 538.0; GVHR: 831.9; WPO_x_ without adjustment: 595.7; WPO_x_ with adjustment: 482.8)

**Figure 2:**
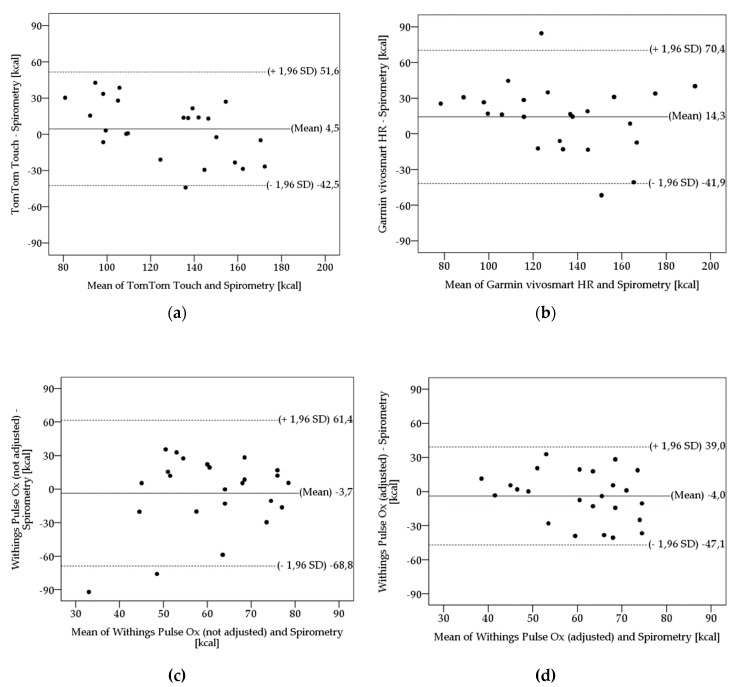
Bland-Altman plots of Spirometry EE for (**a**) TomTom Touch, (**b**) Garmin vivosmart HR, (**c**) Withings Pulse O_x_ (not adjusted), (**d**) Withings Pulse O_x_ (adjusted). Limits of agreement (LoA) were calculated as mean ± 1.96 x SD. Mean biases are depicted as solid line; LoA are depicted as dashed lines.

Figure 2 shows Bland-Altman plots of the tested fitness trackers in comparison with spirometry. These plots serve as a visual illustration of scattering and over- or underestimated measurement ranges of the investigated fitness trackers. 

The plots indicate the differences of the EE values on the y-axis relative to the mean of the two methods (spirometry and alternative method) on the x-axis. Mean differences (bias) between estimated EE and EE of spirometry, upper and lower limits of agreement (ULoA, LLoA) are labeled in the plots. Limits of agreement (LoA) were calculated as means ± 1.96 x SD. The fitness trackers show considerable deviations in scattering, when compared with spirometry. The plots illustrate the TTT’s and GVHR’s tendency to overestimate (bias TTT: 4.5 kcal; bias GVHR: 14.3 kcal) and the WPO_x_’s tendency to underestimate (bias WPO_x_ without adjustment:-3.7 kcal; bias WPO_x_ with adjustment: -4.0 kcal) the EE, respectively. Furthermore, the differences in variance are visualized. The WPO_x_ with adjustment shows the lowest scattering (ULoA-LLoA: 86.1 kcal), whereas the WPO_x_ without adjustment indicates the highest scattering (ULoA-LLoA: 130.2 kcal) amongst its measures. GVHR (ULoA-LLoA: 112.3 kcal) and TTT (ULoA-LLoA: 94.1 kcal) are between the WPO_x_ with and without adjustment.

## 4. Discussion

The present study examined the validity of VO_2max_ and EE estimations of various wrist-worn activity trackers. 

The validity of the devices was determined by four methods. Using the MAPE, systematic differences should be assessed. According to Nelson et al. [6], activity trackers should not exceed a 10% error deviation (MAPE) from the gold standard in order to be considered accurate. The GF920XT achieved this condition (7.3%). The PV800 (13.2%), WPO_x_ with adjustment (14.2%), TTT (18.2%), WPO_x_ without adjustment (20.1%), and the GVHR (23.9%) exhibit greater deviation errors. The results of t-tests comparing estimated VO_2max_ and EE from the activity trackers, respectively, with spirometry indicated that the GF920XT significantly underestimates the VO_2max_ and the GVHR significantly overestimates the EE. The other devices did not show any significant differences in comparison with the gold-standard method. To investigate the level of agreement between the activity trackers and the gold standard, Bland-Altman plots were prepared according to Bland & Altman [33]. Concerning the VO_2max_, the GF920XT reveals a narrower 95% limit of agreement than the PV800 (ULoA-LLoA (GF920XT): 17.2 ml·kg^−1^·min^−1^; ULoA-LLoA (PV800): 30.1 ml·kg^−1^·min^−1^) and therefore visualizes the differences in variance. The plots of EE revealed the narrowest 95% limits of agreement for the WPO_x_ with adjustment (ULoA-LLoA: 86.2 kcal). The WPO_x_ without adjustment indicates the widest 95% limits of agreement with a difference of 130.2 kcal. 

To determine the level of agreement, the ICCs between the activity trackers and the spirometry were examined. Sports watches demonstrate a good (GF920XT) and a moderate (PV800) level of agreement, respectively. The fitness trackers TTT and WPO_x_ with adjustment indicate a moderate agreement with the gold standard. To summarize, one of the activity trackers shows a good level of agreement (GF920XT), and three out of six activity trackers do not (PV800, TTT, WPO_x_ with adjustment). Concerning the validity of the activity trackers, GF920XT and GVHR show significant deviations to the gold standard. Although the other devices indicate no significant differences, they still have considerable deviations in dispersion and measuring range, which should be included in the decision regarding their validity. A lower bias, a lower MAPE, and a better level of agreement of WPO_x_ with adjustment compared with WPO_x_ without adjustment indicate that an additional subjective estimation of the user’s physical strain mostly leads to data that are more accurate. The systematic differences (MAPE) and the range between the limits of agreement of the examined activity trackers are considered to be too substantial. Therefore, their use as an alternative to the gold standard method is questionable. 

Both tested sports watches exceed the absolute error value of 10%, which was suggested by Fokkema et al. [12]. Thus, they must be considered as too inaccurate to recommend them without any concerns regarding their user’s health for general purposes neither in sports, nor in health care and rehabilitative applications. Even though both sports watches seem to be more likely to underestimate an individual’s maximum oxygen uptake, they still sometimes overestimate by a lot. This could lead to harmful situations in one’s health, especially in less experienced users.

Concerning the VO_2max_ estimation of wrist-worn activity trackers, a small number of scientific validation studies have been published so far. To our knowledge, Kraft & Roberts [8] and Snyder et al. [17] are the only scientific validation studies concerning the prediction of the maximum oxygen uptake by means of activity trackers. Kraft & Roberts [8] tested the accuracy of VO_2max_ prediction of the Garmin Forerunner 920XT. Thereby, the sports watch does not show significant differences in comparison with spirometry. Therefore, they estimated the sports watch to be an accurate device to determine the maximum oxygen uptake. This contrasts with the results of the presented study. Although the presented study shows a MAPE ≤ 10% and a good agreement (ICC) in comparison with CM3B, the estimation of the VO_2max_ by the Garmin Forerunner 920XT was not sufficiently valid. This can be justified by significant differences to the spirometry and high internal differences in variance. Kraft & Roberts [8] did not provide any power analysis of their statistics, nor did it calculate systematic differences (MAPE). The author’s assessment regarding the validity of the Garmin Forerunner 920XT exclusively refers to the results of the paired samples t-test. Thus, their conclusions should be considered critically. Snyder et al. [17] investigated the validity of VO_2max_ prediction of three sports watches—Polar V800, Garmin Forerunner 230 and 235. They showed significant differences in comparison with the gold standard. Thus, the authors concluded that these sports watches should be used carefully for exercise prescription. This corresponds to the overall tendency of the present study. However, in detail, the present study shows no significant differences between the VO_2max_ determinations of PV800 and spirometry. Nevertheless, according to a MAPE > 10% and a moderate agreement (ICC) in comparison with CM3B, the PV800 was considered as too inaccurate. Moreover, in Snyder et al. [17], the maximum oxygen uptake estimations differed for men and women. In females, the Polar V800 significantly overestimated the VO_2max_, whereas in males, the PolarV800 significantly underestimated the VO_2max_, respectively. Differences between men and women were not considered in the presented study. However, one weakness of the study of Snyder et al. [17] is the missing analysis of the MAPE values and the missing power analysis. Leboeuf et al. [34] examined the accuracy of maximum oxygen uptake prediction of an in-ear sensor. The analysis of systematic differences between the sensor and the gold standard shows a MAPE of 3.2 ± 7.3%. Therefore, the authors concluded that the in-ear sensor is accurate, despite insufficient statistical confirmation. 

The suitability of fitness trackers for EE estimation in the health sector, e.g. for support of obesity treatment, is not given. Measured values regarding the EE can be used as a rough assessment. If exact values are needed, indirect calorimetry should be preferred. In Evenson et al. [2], a systematic summary of the validity and reliability of activity trackers is provided. This review includes validation studies on activity trackers from the Fitbit and Jawbone brands. For EE, almost all trackers demonstrate good to excellent reliability (ICC = 0.74–0.97). However, they do not provide valid results. Estimation of EE is usually significantly underestimated. Further studies confirm that a lot of wrist-worn activity trackers show insufficient validity and reliability concerning EE [3,4,5,6,7,9,10,11,13,15,16]. Boudreaux et al. [16] investigated the validity of EE estimation during cycling and resistance exercise. Among other devices, they examined the accuracy of the Garmin Vivosmart HR (GVHR) and TomTom Touch (TTT). During resistance exercise, estimation of EE from both fitness trackers had weak intraclass correlation. The GVHR showed the strongest correlation (R = 0.18), whereas the TTT indicated the lowest correlation value (R = 0.02). Additionally, both devices had high MAPE values (GVHR: 57.02%; TTT: 51.64%) during resistance exercise. During cycling, both devices showed high MAPE values (GVHR: 63.05%; TTT: 41.27%) and had weak correlation (GVHR: R = 0.41; TTT: R = 0.30). Based on the t-test, the MAPE, and the ICC, the authors concluded that neither the GVHR nor the TTT represent a valid alternative to the metabolic analyser as the gold-standard method. This conclusion is in accordance with the results of the presented study. However, in detail, there are some differences. The MAPEs of TTT and GVHR show considerably lower values (TTT: 18.2%; GVHR: 23.9%) than in Boudreaux et al. [16]. In addition, the comparison of t-tests indicates different results. In the present study, the TTT does not significantly differ from CM3B, whereas in Boudreaux et al. [16], TTT significantly overestimated EE during the resistance exercise as well as during graded exercise cycling. Exclusively, the GVHR significantly overestimated EE compared with CM3B. Regarding resistance exercise, this is in line with the results of Boudreaux et al. [16]. Because of different study designs, the results of the presented study and the study of Boudreaux et al. [16] are not fully comparable. 

In general, these results substantiate the conclusions of Evenson et al. [2], indicating a low validity for EE estimation in 10 adult studies. Besides the general validity, Wahl et al. [15] examined the influence of running pace on the EE. The study shows a significant influence of running pace on the estimated EE. Energy expenditure tends to be overestimated at lower pace and underestimated at higher pace. To summarize, the authors concluded that most of the tested activity trackers could be assumed as not valid. Equally, Roos et al. [5] examined the validity of estimated EE during running. They concluded that metabolism significantly influences the estimation of EE. In the aerobic range, EE was both over- and underestimated, whereas in the anaerobic range the tested sports watches significantly underestimated EE by 21.6% to 49.3%, respectively. The results of Woodman et al. [3] regarding the accuracy of activity trackers for estimating EE are in accordance with the above mentioned studies [2,15,16]. Almost all of the tested activity trackers showed significant differences from the measured EE. Woodman et al. [3] tested the accuracy of EE estimation of Withings Pulse O_x_, as well. The MAPE was 64%. The MAPE of WPO_x_ found in the present study was considerably lower. MAPE values were 14.2% (with adjustment) and 20.1% (without adjustment), respectively. That variance may be caused by a different study design. In contrast to the present study, Lee et al. [7] examined the validity of EE estimated from a variety of consumer-based activity trackers under free-living conditions. However, there are no differences in the validity of the results. Even in this study, the activity trackers could not show sufficient validity. All tested activity trackers had a mean absolute percentage error of ≥10% compared with the gold standard method, except the BodyMedia FIT. Based on the differing results of the above-mentioned validation studies, estimations of EE and VO_2max_ should be regarded with skepticism and caution.

There were also some limitations to this study. Based on the paired sample t-test, it cannot be conclusively said if the activity trackers are valid, because the values determined in a power analysis exceed the beta error. To reach power levels > 0.8, a sample size of > 30 is recommended. Although the estimations were mostly not significantly different from measured EE and VO_2max_, the activity trackers still show considerable deviations in dispersion and measuring range. This effect can possibly be explained by a low to moderate effect strength. Moreover, an investigation of the reliability of the tested fitness trackers and sports watches by performing repeated sets of measurements should be considered. Another limitation of the present study is the performance under controlled laboratory conditions. Thus, the results can be restrictedly transferred to everyday life. For clarification, it would be useful to conduct a broad field study.

## 5. Conclusions

Activity trackers are a simple, comfortable and mostly low-priced method to capture different physiological parameters. The tested trackers could not show valid results. Hence, it is concluded that current commercially available activity trackers are most likely not accurate enough to be used for neither purposes in sports, nor in health care and rehabilitative applications. Manufacturers need to provide more information about the accuracy of their devices, as well as to improve the performances to make them eligible for use. 

## Figures and Tables

**Table 1:** Physical characteristics of participants. Values are mean ± SD (range).

	Male (*N* = 13)	Female (*N* = 11)	All Participants (*N* = 24)
Age (yr)	22.6 ± 1.0 (21.0–24.7)	24.4 ± 2.6 (21.1–29.8)	23.4 ± 2.1 (21.0–29.8)
Height (cm)	183.8 ± 4.4 (178.0–191.0)	166.7 ± 5.6 (157.0–176.0)	176.0 ± 10.0 (157.0–191.0)
Weight (kg)	81.6 ± 8.8 (67.3–104.0)	61.1 ± 11.1 (51.6–80.9)	72.2 ± 14.2 (51.6–104.0)
BMI ^1^ (kg∙m^−2^)	24.2 ± 2.2 (19.7–28.5)	21.9 ± 3.0 (19.4–27.9)	23.1 ± 2.8 (19.4–28.5)

^1^ Body mass index.

**Table 2:** Descriptive examination of the differences between the measured VO_2max_ (CM3B) and the estimated VO_2max_ of the investigated sports watches. Arithmetic mean of estimated VO_2max_ ± standard deviation (Mean ± SD); mean absolute error ± standard deviation (MAE ± SD); mean absolute percentage error (MAPE); intraclass correlation coefficient (ICC); test statistic (t); probability value (*p*); statistical power (*P*).

	N	Mean ± SD (ml·kg^−1^·min^−1^)	MAE ± SD (ml·kg^−1^·min^−1^)	MAPE	ICC	t	*p*	*P*
PV800	24	3.0 ± 7.7	6.5 ± 4.7	13.2	0.67	1.89	0.071	0.44
GF920XT	24	–2.1 ± 4.4 *	3.8 ± 2.9	7.3	0.82	–2.37	0.027	0.62

* Significantly different from CM3B (*p* < 0.05).

**Table 3:** Descriptive examination of the differences between the measured EE (CM3B) and the estimated EE of the investigated fitness trackers. Arithmetic mean of estimated EE ± standard deviation (Mean ± SD); mean absolute error ± standard deviation (MAE ± SD); mean absolute percentage error (MAPE); intraclass correlation coefficient (ICC); test statistic (t); probability value (*p*); statistical power (*P*).

	N	Mean ± SD (kcal)	MAE ± SD (kcal)	MAPE (%)	ICC	t	*p*	*P*
TTT	24	4.5 ± 24.0	20.1 ± 12.9	18.2	0.79	0.93	0.363	0.14
GVHR	24	14.3 ± 28.7 *	26.3 ± 17.3	23.9	0.71	2.44	0.023	0.65
WPOx not adj.^1^	24	–3.7 ± 33.2	24.3 ± 21.9	20.1	0.62	–0.54	0.590	0.08
WPOx adj.^1^	24	–4.0 ± 22.0	17.7 ± 13.0	14.2	0.84	0.90	0.377	0.14

^1^ adj., adjusted. * Significantly different from CM3B (*p* < 0.05).
